# The Evolution of Public Health Genomics: Exploring Its Past, Present, and Future

**DOI:** 10.3389/fpubh.2018.00247

**Published:** 2018-09-04

**Authors:** Caron M. Molster, Faye L. Bowman, Gemma A. Bilkey, Angela S. Cho, Belinda L. Burns, Kristen J. Nowak, Hugh J. S. Dawkins

**Affiliations:** ^1^Office of Population Health Genomics, Public and Aboriginal Health Division, Western Australian Department of Health, East Perth, WA, Australia; ^2^Office of the Chief Health Officer, Public and Aboriginal Health Division, Western Australian Department of Health, East Perth, WA, Australia; ^3^School of Biomedical Sciences, Faculty of Health and Medical Sciences, University of Western Australia, Crawley, WA, Australia; ^4^Harry Perkins Institute of Medical Research, QEII Medical Centre, Nedlands, WA, Australia; ^5^Sir Walter Murdoch School of Policy and International Affairs, Murdoch University, Murdoch, WA, Australia; ^6^School of Public Health, Curtin University of Technology, Bentley, WA, Australia

**Keywords:** public health genomics, precision public health, genomics, population genetics, population health

## Abstract

Public health genomics has evolved to responsibly integrate advancements in genomics into the fields of personalized medicine and public health. Appropriate, effective and sustainable integration of genomics into healthcare requires an organized approach. This paper outlines the history that led to the emergence of public health genomics as a distinguishable field. In addition, a range of activities are described that illustrate how genomics can be incorporated into public health practice. Finally, it presents the evolution of public health genomics into the new era of “precision public health.”

## Public health: the past

The field of public health emerged as a means to “protect” the health of the individual and the community, and thereby minimize morbidity and mortality associated with disease (1, 2). Differentiating itself from the medical field, public health places an emphasis on improving the health of society as a whole through the use of organized, population-wide approaches. Instances of public health efforts have been documented throughout history. For example, by the eighteenth century, it was common practice to isolate and quarantine sick individuals, in order to contain the spread of contagious diseases such as leprosy and the plague (1, 3). Developing and delivering appropriate public health services requires an understanding about health and wellbeing, the presence and absence of disease, how health outcomes are distributed within populations and the factors that determine these outcomes.

Over the last two centuries, the essential activities of public health have evolved significantly. In the nineteenth century, the primary focus of public health was on managing the physical environment, such as the provision of safe drinking water and the development of effective sewerage systems. In the twentieth century, the scope of public health was increased to include social factors (such as housing, employment, income, educational level, and access to transportation and health care) and lifestyle behaviors (such as physical activity, diet, smoking, and alcohol consumption) that are now known to influence health outcomes. This led to the emergence of the “health promotion” era of public health, which stemmed from a movement aimed at providing evidence-based education that would enable people to increase control over and improve their health (4). The health promotion movement drove action in a range of areas of public health including: developing public policy, creating environments that support healthy behaviors, and empowering people to develop personal skills to make choices that lead to healthier lives.

At the heart of public health today is the recognition that health outcomes are influenced by a range of social, cultural, political, economic, environmental, behavioral, and biological (e.g., genetic) factors (5). Otherwise known as the “determinants of health,” these factors may favor health or be harmful to it. Further, while some factors cannot be changed (such as age or ethnic background), others may be modifiable (for example, weight or smoking status). Understanding how these factors influence health outcomes is key to informing public health approaches for promoting health and wellbeing, through the implementation of practices that aim to “prevent” poor health. These prevention strategies can be categorized into three levels, being:

- **Primary:** where the aim is to prevent disease and injury from occurring, which will reduce their incidence in the population. This is done largely through interventions to eliminate risk factors. For example, seatbelts, sunscreen, tobacco-use cessation.
- **Secondary:** where the aim is to reduce the more immediate impact of disease and injury if it does occur. The focus of interventions is on early detection and treatment to alter or slow progress of the disease or injury, and thereby prevent the onset of long-term or permanent adverse consequences such as complications and disabilities. For example, population screening programs.
- **Tertiary:** where the aim is to help people manage the longer term impact of ongoing disease or injury. The focus of interventions is to improve, as much as possible, factors such as physical and mental functioning, quality of life, and life expectancy. For example, chemotherapy, rehabilitation.

A fourth category of prevention, known as quaternary prevention, has also been proposed (6, 7). This is defined as: “action taken to identify a patient or a population at risk of over-medicalization, to protect them from invasive medical interventions and provide for them care procedures which are ethically acceptable” [(6), p. 3]. In other words, the aim of quaternary prevention is to identify and protect those individuals for whom medical interventions are likely to cause more harm than good (7, 8).

## Core functions of public health

Following from the health promotion movement was a growing recognition that public health had changed significantly over the years, and that the governmental role in providing public health services needed to be clearly defined, adequately supported, and fully understood. This led the Institute of Medicine (IOM; now known as the Health and Medicine Division) of the United States of America's National Academies of Sciences, Engineering and Medicine in 1988 to identify and define the three core functions to be provided by all public health agencies (3), being:

- **Assessment:** to assess and monitor the health of communities and populations at risk, and to identify health problems and priorities. This requires the regular and systematic collection, assembly, analysis, and dissemination of information on the health of populations.
- **Policy development:** to formulate public policies, plans, standards, guidelines, and resources in collaboration and partnership with stakeholders, and to solve identified local and national health problems and priorities.
- **Assurance:** to assure that all populations have access to appropriate and cost effective care (including health promotion and disease prevention services), and to evaluate the effectiveness of healthcare and public health interventions.

Building on this work, in 1994 the three core functions of public health were further elaborated into 10 essential public health services, to support the application of the core functions in practice (9, 10). These 10 essential public health services are presented in Box 1.

Box 1 **The 10 essential public health services (9).**

1. Monitor and evaluate health status to identify community health problems.
2. Diagnose and investigate health problems and health hazards in the community.
3. Inform, educate, and empower people about health issues.
4. Mobilize community partnerships to identify and solve health problems.
5. Develop policies and plans that support individual and community health efforts.
6. Enforce laws and regulations that protect health and ensure safety.
7. Link people to needed personal health services and assure the provision of health care when otherwise unavailable.
8. Assure a competent public and personal health care workforce.
9. Evaluate effectiveness, accessibility, and quality of personal and population-based health services.
10. Research for new insights and innovative solutions to health problems.

## Public health genomics: the present

In the two decades since the core functions and essential services of public health were defined, rapid developments have occurred in the field of genomics (see Box 2 to understand the distinction between the terms “genetics” and “genomics”). These developments have enhanced our knowledge of how human genes interact with each other and the environment to influence health. A notable example is the completion of the Human Genome Project in 2003, which led to the proliferation, in volume and complexity, of knowledge about the human genome. It also resulted in a significant reduction in the estimated number of genes expected to be found in the human genome, down from previous estimates of as many as 140,000 genes to a probable 20,500 (13). The impact that advancements in genomics have had on our understanding of disease is discussed in Box 3.

Box 2 **The difference between genetics and genomics.**

Genetics is the science of inheritance and tends to look at the functioning and composition of a single gene at a time. Thus genetic studies into diseases tend to focus on those that are associated with variants in one gene only (11). These “single gene disorders” (or Mendelian disorders) are relatively rare in the population and examples include Fragile X syndrome, cystic fibrosis, muscular dystrophy, and Huntington's disease.

Most common diseases are multi-factorial, caused by variants in numerous genes that interact with each other and with a range of environmental factors (12). To gain more knowledge about these diseases, researchers work in the field of genomics. This field involves the study of the genome, that is, all the genes in the cells of an organism and how these genes interact with each other and with environmental factors to affect an organism's growth and development (11). Hence genomics researchers are able to explore the causes of diseases such as cancer, diabetes, and heart disease, which have multi-factorial determinants including genes, lifestyle behaviors, and other environmental influences.

Box 3 **How genomics improved the understanding of diseases.**

“Genomic knowledge” refers to the information that is obtained from studying the complete genetic makeup of a cell or organism. In recent years, scientific research in this area has contributed significantly to our knowledge about the human genome, improving our ability to understand disease etiology, risk, prevention, diagnosis, and treatment. The ways in which these areas can be enhanced by genomic knowledge are outlined below. Based on these improved understandings, genomic tools, and technologies are being developed to enable better health not just for the individual, but for populations as well.

***Etiology***

Increased genomic knowledge about a disease can provide insights into how the disease may develop. This can occur through a better understanding of the function of genes that make up the genome, how different genetic variants contribute to the phenotype of diseases, the role of gene expression, and the role of the interaction between genes (10, 14).

***Risk***

Genomic knowledge is expected to improve our understanding of why some individuals remain healthy while others are more susceptible to disease. For example, information on the genetic variants associated with an increased risk of common diseases, such as cardiovascular disease and diabetes, might at some point be used to make predictions about the likelihood a person will get these diseases (15). This knowledge could then be applied to develop new tools for risk prediction or predictive testing (5) in relation to the onset or recurrence of disease (16).

***Prevention***

Understanding how the genome influences the etiology and risk of diseases may lead to improved understanding of how diseases, or the symptoms of disease, can be prevented (5, 16). Genomic tools and technologies can also identify infectious diseases with greater speed and precision to enable rapid responses to disease outbreaks and more efficient surveillance (17–19).

***Diagnosis***

Historically, clinicians generally used a set of observable or measurable characteristics as the basis for diagnosing disease. Genomic knowledge takes this one step further, by enabling clinicians to look at a person's genes to provide a molecular diagnosis. In line with this, diagnostic technologies have been developed that include a plethora of clinical genetic tests (5, 14, 16).

***Treatment***

To date, genomic knowledge has mostly been used to inform disease treatments. Pharmacogenetics and pharmacogenomics are two fields where new and improved therapies and treatments have been developed, including hundreds of new drugs which are advancing disease management (16). The expectation is that genomic knowledge will further improve the ability to assess treatment responses, such as how different people metabolize drugs and which people are more likely to experience adverse drug reactions (11, 16, 20). Based on genetic profiles, tailored therapies may be developed for an individual and across individuals within specific patient populations to deliver the right drug in the right dose at the right time (12, 16, 21, 22).

From these advancements comes the increasing recognition of the potential applications of genomic knowledge and related technologies to improve population health. For example, genomic knowledge can offer new ways of differentiating individuals and sub-groups within populations, taking public health beyond the traditional correlates of disease risk such as gender, age, and socio-economic status (20). Specifically, it can enable the stratification and subsequent screening of individuals and sub-groups of populations based on their level of genetic risk for developing a disease. This can then lead to the development of more targeted prevention approaches to reduce the burden of disease (11).

It should be noted that advances in genomics have been dependent on, and facilitated by, progress in related fields such as informatics, and the development of novel technologies capable of evolving to meet the increasing demands of genomic medicine. Specifically, the huge volume of data generated by next generation sequencing has created significant challenges relating to data storage and analysis. These challenges are explored further in Box 4.

Box 4 **The data informatics puzzle.**

The concept of Moore's Law is useful to consider, when exploring the limitations of current computation and storage in genomics medicine. Moore's Law was proposed in 1965 to describe the long term trend whereby for every doubling of time of ~18 months, there is an exponential increase in the capacity for disk storage and computation (23). Historically, this growth meant that data storage and computation were able to stay ahead of the demands of genomics. However, the advent of next generation sequencing in 2005 and the rapid decline in associated costs have resulted in demands on the informatics capacity outpacing developments in the informatics ecosystem (24). The 100,000 Genomes Project in the United Kingdom (UK) has highlighted the limitations of digital infrastructure in the progression of genomic medicine, and the UK government has committed to fund sufficient digital infrastructure in order achieve successful rollout of their Genomic Medicine Service (25). Future integration of genomics in population medicine must emphasize the development of sustainable computational analytics and storage infrastructure.

Many tools and technologies based on emerging genomic knowledge have been developed. However, for a range of complex reasons, only a small proportion of these tools and technologies have so far been fully translated into healthcare and public health practice from the discovery research phase, beyond the introduction of newborn screening for genetic conditions (20, 26). The literature refers to two key reasons why this may be so. Firstly, in genomic studies, most genetic variants that have been identified as contributing to common diseases are only associated with small increases in relative risk and explain only a little about the relationship between disease and genetic inheritance (10, 21, 27, 28). This is because most common diseases are the result of complex interactions between multiple genes and environmental factors. Furthermore, the genetic variants that contribute to a given disease, and how they are expressed, may vary among different people and sub-populations, as might the relative significance of genetic and non-genetic factors (12). Consequently, attention has shifted toward rare and monogenic diseases where the gene and phenotype(s) may result in more clear causal pathologies. Nevertheless, obtaining a definitive association between a single-gene variant and a distinct disease phenotype remains a complex process.

Secondly, while tools and techniques based on genomic knowledge have been developed, there has often been limited evidence regarding their validity and utility (26). In part, this is due to a lack of investment in the infrastructure required to collect and evaluate tools and technologies in a systematic manner (10, 29) and also to the complexity of conducting evaluations (27). The recognition of this lack of evidence gave rise to the discipline of “public health genomics,” defined in 2005 as “the responsible and effective translation of genome-based knowledge and technologies for the benefit of the population” (30).

While there are expectations that genomic knowledge, tools, and technologies benefit population health, it is essential that they are applied only when the benefits outweigh the potential harms. New tools and technologies that are prematurely introduced without the evidence demonstrating that they are valid and useful run the risk of posing harm to individuals, families, and the broader health system. Such issues might include the potential for over-, under-, or mis-diagnoses, or psychosocial harms. It is also critical to consider the ethical, legal, and social issues inherent in the field of genomics. These issues are particularly relevant in the context of genomic information relative to other medical information due to the fact that variants in genes, by nature, are shared within families. Uncovering genomic determinants of health therefore has implications not only for the individual but for genetic relatives as well. Moreover, genomic information can be obtained in the absence of clinical symptoms and therefore in isolation it may have a weaker predictive association with health outcomes compared to most other health information. In addition, determining who, what, and when to test is fraught with ramifications for service capacity and financial responsibility, and can also have implications for patient autonomy and privacy as evident in the case study presented in Box 5.

Box 5 **case study—ethical, legal, and social implications to consider for applications of public health genomics.**

Consider the hypothetical scenario in which a newborn screening program performs whole genome sequencing on every newborn within a population. The ethical, legal, and social implications to consider include:

- Which variants should be reported? Should they be limited to known pathogenic variants, or further limited to only those that have an available treatment? Should variants of unknown significance be reported?
- Who decides what information, such as variants of unknown significance or secondary findings, should be reported? Should this be a decision for parents, or for an independent governance body?
- Which conditions should be screened for? Would parents want to know their baby's risk of developing a late-onset disorder such as dementia, or an untreatable condition? Would a child want to know of their risk?
- How should genetic counseling be offered to all parents of newborns such that they can give informed consent for the tests?
- Does the population have sufficient genetic literacy to be able to fully understand the consent process, and implications of the results, for benign, pathogenic, and uncertain variants?
- Should the genomic data be kept, and if so, for how long?
- Should the data be re-interrogated, particularly with inevitable advances in technology? If so, at what time intervals?
- Should these genomic data be available to all healthcare providers?
- What are the implications on health and life insurance if disease risk can be stratified at birth?
- If a baby is shown to be stratified at higher risk for certain lifestyle diseases, what is their responsibility for mitigating that risk? What is the government's responsibility for mitigating that risk?

It is therefore essential to consider existing and emerging knowledge, tools, and technologies in order to determine which are actually beneficial to population health and how they could be appropriately implemented. This requires an objective evaluation of the potential benefits against the potential harms, and the resources required for implementing them (12). Public health genomics bridges this gap between new scientific discoveries and technologies, and the application of genomic knowledge to benefit population health (31, 32).

With genomics being increasingly integrated into population-level health initiatives, it has been internationally recognized that maintaining efficiency, effectiveness, ethics, and equity into the future requires a strategic approach. In line with this, there has been a call for the cooperative development and harmonization of policy on genomics in healthcare between 28 of the European Union member states and Norway (33). Of these nations, Italy has been a leader in the development of public health genomics policy, developing a National Plan for Public Health Genomics that includes consideration of translation of genomics into public health practice (34). An international working group on “Beyond Health Genomics” also recommended the improved facilitation of translation research through greater engagement between public health professionals, geneticists, and scientists (20).

Similarly, the Australian Government's Department of Health has released the *National Health Genomics Policy Framework 2018–2021* to harness the health benefits of genomic knowledge and technology into the Australian health system. This framework provides a shared direction and commitment between all governments in Australia to consistently and strategically integrate genomics into the Australian health system through five strategic priority areas: person-centered approach, workforce, financing, services, and data (35). The cohesive strategy is expected to ensure that the integration of genomics in healthcare is not only appropriate for the health of populations, but is also sustainable for the health system.

## Public health genomics in practice

The ways in which genomics can contribute in public health practice are clear. However, capitalizing on genomic advances requires a coordinated approach in order to integrate the benefits of associated knowledge and technologies into each aspect of public health service delivery. Beskow et al. (31) were the first to link the 10 essential public health services—provided in Box 1—with genomics, in 2001. Integral to each essential service is the role of “system management” in ensuring the responsible, equitable, and sustainable integration of genomics into healthcare and public health practice.

Almost 20 years after the link between public health and genomics was established, Table 1 furthers Beskow et al.'s. (31) work to provide examples from the literature of how genomics can be incorporated into public health practice. These examples reflect the rapid developments made in genomics and the significant impact the field has had to improve population health.

**Table 1:** Public health genomics activities in relation to the 10 essential public health services.

**Essential public health services**	**Public health genomics activities**
1. Monitor health status to identify and solve community problems	**Assess the distribution and impact of modifiable and genetic risk factors to determine their contribution to health status and the burden of disease** (31, 32). A better understanding of these risk factors could enable more precise decision-making about resource allocation and the prioritization and targeting of public health programs, and lead to new approaches to disease prevention and treatment (31).
	**Promote the development of resources that enable monitoring of the genomic-related health status of populations**. Key activities could include (5, 11, 36, 37): assessing the inclusion of genomics information in the collection, management, and analysis of routine data working with national surveys and large epidemiology groups to maximize potential from databases exploring the potential for disease-specific, and population-based, registries to be used to conduct disease surveillance.
2. Diagnose and investigate health problems and hazards in the community	**Identify and track infectious disease outbreaks using genomic technology** This involves utilizing genomic technology to improve the speed and efficiency of infectious disease surveillance and response (17–19).
	**Assist with the redesign of diagnostic and laboratory services to incorporate new genome-based technologies** (38). Examples of these technologies include massively parallel sequencing such as whole exome and whole genome sequencing (39). There is potential for the incorporation of these technologies into diagnostic and laboratory services that can improve the diagnostic yield from genetic testing.
3. Inform, educate, and empower people about health issues	**Improve the genomic literacy of the public** (22, 31, 37). This involves providing education materials to communities that teaches them about genetics and genomics in understandable language (37, 39–43).
	**Empower all stakeholders, including health professionals and the public, to make informed decisions about the uses of genetic information with realistic expectations about the risks and benefits** (31). This includes the provision of relevant information on the uses of genomic information in disease prevention (22, 31), as well as on the associated ethical, legal and social issues.
	**Facilitate the integration of genomics into health promotion and disease prevention programs** (31). This will contribute to informing and educating people about genomics knowledge and technologies, as well as its limitations.
4. Mobilize community partnerships to identify and solve health problems	**Foster collaborations between stakeholders** (31). This encompasses capacity building, and developing networks and partnerships between diverse stakeholders including public policy makers, patients, the general public, academia, clinicians, researchers, and industry (16).
5. Develop policies and plans that support individual and community health efforts	Policies and plans that could be developed include those relating to: the **appropriate use of genomic applications** (33, 37), through standards and guidelines that recognize the complexity of genomics and define when and how genome-based information and technologies should be used to promote health and prevent disease (31, 34, 44), including in the clinical setting (36, 45, 46) **equity and accessibility**, to assure genomics knowledge and technologies are accessible across all segments of the population (20, 37) the **use of family health history information** to inform people of the role of inheritance in the development of disease and identify people at risk of disease (26, 37) **reproductive decision-making**, including prenatal screening, population-based carrier screening and pre-implantation genetic diagnosis (22).
6. Enforce laws and regulations that protect health and ensure safety	Contribute to: **laws and regulations for genomic applications** (37). This could apply to genetic tests, including direct-to-consumer tests and related issues such as funding, data protection, insurance coverage for high-risk individuals and the prevention of genetic discrimination (22, 37, 42, 47). **regulations for laboratories using genome-based technologies** (22). An example of these technologies is massively parallel sequencing.
7. Link people to needed health services and assure provision	**Support the appropriate integration of genomic knowledge and technologies into all aspects of healthcare and public health** (26, 41, 48). This may be operationalized in a number of ways, such as: supporting the implementation of evidence-based genomic applications and discouraging the use of unvalidated applications (32), to prevent the premature use, misuse and overuse of genomic applications (11). providing expert advice on the commissioning of services that use genome-based knowledge and technologies (38). This may relate to issues such as the appropriateness of the technologies for use; and the impact on, or requirements for, supporting functions such as counseling, education, and service coordination (45). supporting the incorporation of genomic applications into existing public health practice, such as: using pathogen and human genomic technologies to control and manage communicable diseases (16); expanding population-based screening programs to include the use of genetic information (41); and targeting interventions for preventing diseases in population groups based on genetic information (11). promoting the use of family health history to identify individuals at risk of disease (37, 40). Family history is the most consistent risk factor for all diseases and reflects the complex interactions between genes, behaviors, cultures and environments that family members share (49). It can be used to identify families at high risk for disease and could be incorporated into tailored chronic disease prevention and health promotion messages (40). ensuring equity and accessibility to genomic applications and services (29, 36, 37, 42). This is especially important for population groups that traditionally face barriers to accessing health services, such as Indigenous and low socio-economic groups (31).
8. Assure a competent public and personal healthcare workforce	**Contribute to training and education in, and development of, genomic knowledge, skills and capacity for health professionals** (31, 43, 45, 50). This is so that: genomics is appropriately integrated into their work; they can effectively communicate genetic information; and they can support informed decision-making by patients (51).
	**Support the development of workforce capacity in genomics-related fields**. These fields include bioinformatics, genetic epidemiology, law and ethics, and health economics as applied to genetics and genomics (16, 38, 52, 53).
9. Evaluate the effectiveness, accessibility and quality of health services	**Evaluate new genome-based knowledge and technologies to determine their evidence base, quality, appropriateness and readiness for implementation in healthcare and public health practice**. The need for evaluation is based on concerns that the availability of genome-based tools and technologies, such as genetic tests, diagnostic equipment and therapies, are being driven more by technical feasibility and commercial potential than by evidence-based implementation. Such evaluations ensure that the benefits of genomic discoveries are realized efficiently, effectively and equitably, and are only implemented when it is in the public's best interest (2, 5, 27, 29, 31, 32, 42, 45, 54).
	**Evaluate the use of genome-based knowledge and technologies in healthcare and public health practice** (11, 55). Examples of evaluations include: the current use of genetic tests and services; the factors that influence utilization; cost-effectiveness; and the impact on service, intervention and patient outcomes (11, 20, 31, 36, 56).
10. Research for new insights and innovative solutions to health problems	**Monitor the results of human genome epidemiology studies** (45). This provides a population perspective on gene-disease associations, estimating the contribution of gene variants to the occurrence of disease in groups and the population overall (31, 37, 44, 46). Monitoring these studies can help identify gaps in knowledge at the population level (11) and could lead to changes in public health prevention interventions and disease management (14, 44).
	**Support the development of infrastructure for conducting genomic-related population research**. Patient registries, population data sets and linked biobanks are key resources enabling the conduct of large population studies to assess gene-environment interactions (14). However, steps must be taken to ensure that databases reflect genomic reference ranges for the whole population, inclusive of minority groups, to avoid inequity of the applications of genomic technology and knowledge (57).
	**Conduct and monitor translation research** (20, 37). The aim of translation research is to move appropriate genomic technologies from the discovery phase to application in healthcare and public health practice, and to evaluate its use in practice for improving health outcomes (11, 58, 59).

It should be noted that the ability of individuals to directly access health-related genetic and genomic tests, otherwise referred to as direct-to-consumer (DTC) or “personal genomic” tests, is one particular issue for public health that requires consideration. DTC tests may detect individuals at increased risk of certain diseases. However, the clinical utility and validity of DTC tests is largely uncertain (60–63). Furthermore, there are a range of ethical, legal, and social issues associated with such tests, such as challenges relating to the provision of information about the test and associated results, and obtaining informed consent (61, 64). Given that consumers are able to access some tests without clinical oversight, appropriate regulatory mechanisms need to be implemented to ensure public access to such tests is appropriate and that where possible, results are interpreted and communicated with caution (65). For those individuals with results of clinical significance, quaternary prevention principles should be applied to avoid their over-medicalization, particularly where results are uncertain or not based on evidence (63, 66, 67). Also for consideration is the possibility of under-medicalization of individuals if their genomic results are inappropriately interpreted or actioned.

## Precision public health: the future

The integration of genomic knowledge and technologies into healthcare is revolutionizing the way we approach clinical and public health practice. In clinical practice, advances in genomics are allowing information about an individual's genetic and biochemical composition, as determined by the interactions between their genes, environment, and lifestyle, to be used in the delivery of targeted interventions; a field known as “precision medicine” (68). This then enables clinicians to tailor medical treatments better suited to the genetic composition of their patient.

An example of a current initiative that is anticipated to have significant implications for advancing precision medicine is the 100,000 Genomes Project in the United Kingdom, which is briefly discussed in Box 4. This project is sequencing genomes from people with a rare disease and their families, and patients with cancer, in order to improve diagnosis, treatment and care (69). Additionally, in the United States of America, the National Institutes of Health's “All of Us” research program aims to sequence at least 1 million Americans and analyse their health data (70). Launched as part of the US Precision Medicine Initiative, the program will gather environmental and biological information from participants to facilitate and advance research, technology, policies, and individualized medical care (71). The program presents a number of ethical, legal, and social challenges (72) and will serve as a guide for future precision medicine initiatives.

Parallel to the developments in precision medicine has been the advancement of technologies that enable the production, aggregation, analysis, and dissemination of extremely large volumes of individual- and population-level data on genes, environment, behavior, and other social and economic determinants of health. These data have proven useful in finding new correlations, patterns and trends, particularly those involving complex interactions, in relation to diseases, pathogens, exposures, behaviors, susceptibility (risk), and health outcomes in populations (73–75). These technologies and data, such as massively parallel sequencing and genomic reference databases, are now being further utilized to complement and extend the vision of precision medicine, to consider how they can be used to improve health outcomes at the population level (74, 76). This emerging field, of utilizing big data to guide the right intervention to the right people at the right time, has been termed “precision public health” (77). Another way precision public health has been defined is as “the application and combination of new and existing technologies, which more precisely describe and analyse individuals and their environment over the life course, in order to tailor preventive interventions for at-risk groups and improve the overall health of a population” (78).

Building upon the work of public health genomics from the last 20 years, precision public health enables the integration of genomics into public health strategies within the wider context of other determinants of health, such as socioeconomic, behavioral, and environmental factors. This can then lead to more precise individual and population-based interventions (74, 77, 79), and ultimately, improve population health outcomes (78).

The ways in which public health interventions and activities may become more “precise” as a result of technological innovations and the data they produce are evident in a number of areas including: epidemiology; knowledge of the determinants of health; targeting of healthcare disparities; screening and prevention; diagnosis; surveillance; and response to communicable diseases (10, 29, 73, 74, 76, 79–81). For example, genomic technologies could be applied using a precision public health approach to identify the impact of genomic variants in different population subgroups. Each subgroup could then be targeted with tailored interventions that are more relevant to their level of risk, resulting in more efficient and effective disease prevention, screening, and surveillance strategies. Such work is critical given current recognition that a lack of appropriate reference data for ancestral population subgroups could be contributing to disparities in access to effective health interventions (82). This is more likely to occur in minority or disadvantaged populations because they are commonly underrepresented in genomic research (82, 83). Consideration of genetic diversity helps to prevent the misclassification of benign genetic variants as pathogenic for these subgroups, and vice versa, which may otherwise lead to inappropriate care and management (84).

## Conclusion

Public health genomics has been successfully integrated into existing paradigms for the provision of traditional public health services. The continued alignment of genomics with public health promises to deliver more precise, personalized health care to benefit the population. Governments and policy makers in this arena have a unique role to play in guiding this activity in such a way that ensures effective and equitable implementation of genomic knowledge and technologies into health systems. A national, coordinated approach to provide centralized governance of decision-making is required to ensure responsible delivery, universality, and equity of access. In addition, investment in important enabling infrastructure such as data informatics and a genomics-literate workforce will be critical to the sustainability of public health genomics and will prepare health systems to reap the valuable benefits of precision public health.

## Author contributions

All authors contributed substantially to the conception of the work and have given final approval for the manuscript to be published. CM and FB drafted the manuscript. GB, AC, BB, KN, and HD revised the manuscript and provided critical feedback.

### Conflict of interest statement

The authors declare that the research was conducted in the absence of any commercial or financial relationships that could be construed as a potential conflict of interest.

## Glossary

**Table:**

Determinants of health	All the factors that determine health and wellbeing outcomes, including the presence or absence of disease.
DNA sequence	The linear order of the four bases of DNA, that is, the nucleotides called adenine, guanine, cytosine, and thymine.
Epidemiology	The study of the patterns, causes, and effects of health and disease in populations.
Gene	A defined unit of DNA (made up of a DNA sequence) that is inherited and provides instructions that determine characteristics of offspring.
Gene expression	The process by which information from a gene is converted into instructions that are used to create a functional gene product (e.g., a protein).
Genetic variant	A difference in the DNA sequence that makes up a gene. Genetic variations are what make each person unique.
Genetics	The science of inheritance, which generally focuses on one gene at a time.
Genome	All the genes within an organism.
Genome-based knowledge	Facts and information that are acquired through studies in “omics” fields such as genomics, proteomics, and metabolomics.
Genomics	The study of the genome, how all the genes in the genome function and are expressed, and how they interact with each other and the environment to affect an organism's growth and development.
Genomics knowledge	Facts and information that are acquired through the study of the genome.
Genomics technology	The collection of techniques, tools, methods, processes and tests that are developed based on knowledge of the genome.
Genotype	The full set of an organism's genetic variants that make up their unique personal genome.
Health disparities	Differences in the health status of different groups of people, including differences in the incidence and mortality of specific diseases.
Human genome epidemiology	The application of epidemiology approaches to understanding the impact of the human genome on patterns, causes and effects of health and disease in populations. This involves exploring the role of the genome and its interaction with environmental factors to contribute to health and disease.
Incidence	The number of new cases of a disease in a population within a given time period.
Interventions	Activities that aim to reduce risks or threats to health.
Massively parallel sequencing	An approach to DNA sequencing (the process of establishing the exact order of nucleotides within a sample of DNA), which is used to test for and diagnose genetic disorders.
Morbidity	The existence of a disease and the degree to which it affects a person, which can be measured by the incidence of ill health in the population.
Mortality	The number of deaths within a population.
Pharmacogenetics	The study of how variation in a single gene influences a person's response to a drug.
Pharmacogenomics	The study of how the full set of a person's genes (genome) affects their response to a drug.
Phenotype	The observable characteristics or traits of an organism, which is influenced by both genotype and the environment.
Precision public health	The application and combination of new and existing technologies, which more precisely describe and analyse individuals and their environment over the life course, in order to tailor preventive interventions for at-risk groups and improve the overall health of a population.
Prevalence	The number of people in a population who are alive with a disease during a period of time (period prevalence) or at a particular date in time (point prevalence).
